# Synchronous Fluorescence as a Green and Selective Method for the Simultaneous Determination of Cetirizine and Azelastine in Aqueous Humor

**DOI:** 10.1007/s10895-022-02913-6

**Published:** 2022-03-28

**Authors:** Walaa Nabil Abd-AlGhafar, F. A. Aly, Zeinab A. Sheribah, Samar Saad

**Affiliations:** Faculty of Pharmacy, Pharmaceutical Analytical Chemistry Department, Mansoura University, Mansoura, 35516 Egypt

**Keywords:** Cetirizine, Azelastine, Synchronous Fluorimetry, Eye Drops, Aqueous humor

## Abstract

A green, simple, quick and economical method is implemented for the first time for the simultaneous estimation of cetirizine (CTZ) and azelastine (AZE) as co-administered eye drops. The method relies on synchronous spectrofluorimetry with ∆λ = 60 nm. Cetirizine can be estimated at 231 nm and AZE can be measured at 294 nm, each at the other’s zero crossing point. All factors affecting the method were studied and properly optimized. Good correlation was obtained in the range of 0.1–2 µg mL^−1^ for both drugs. The limits of detection were 0.014 and 0.010 µg mL^−1^ and limits of quantitation were 0.043 and 0.029 µg mL^−1^ for CTZ and AZE, respectively. Moreover, ICH guidelines were carried out to validate the adopted method. The method was suitable for the analysis of CTZ and AZE in synthetic mixtures, eye drops and aqueous humor. The mean percentage of recoveries of CTZ and AZE in spiked aqueous humor were 99.83 and 99.37, respectively. Furthermore, Green Analytical Procedure Index (GAPI) and analytical Eco-scale approaches were used to evaluate the greenness of the suggested method.

## Introduction

Ocular allergy disease has become more prevalent during the last several decades as it affects 40% of the population globally [1]. It causes significant decline in the work and educational productivity as well as the overall quality of life [2]. Seasonal and perennial allergic conjunctivitis are the most common types of ocular allergy, in which the conjunctiva of the eye becomes inflamed as a result of an immunoglobulin E-mediated hypersensitivity reaction [3]. Ocular allergy results from pollen, molds and dust mites leading to watery eyes, itching, irritated eye and chemosis [4].

**Fig. 1:**
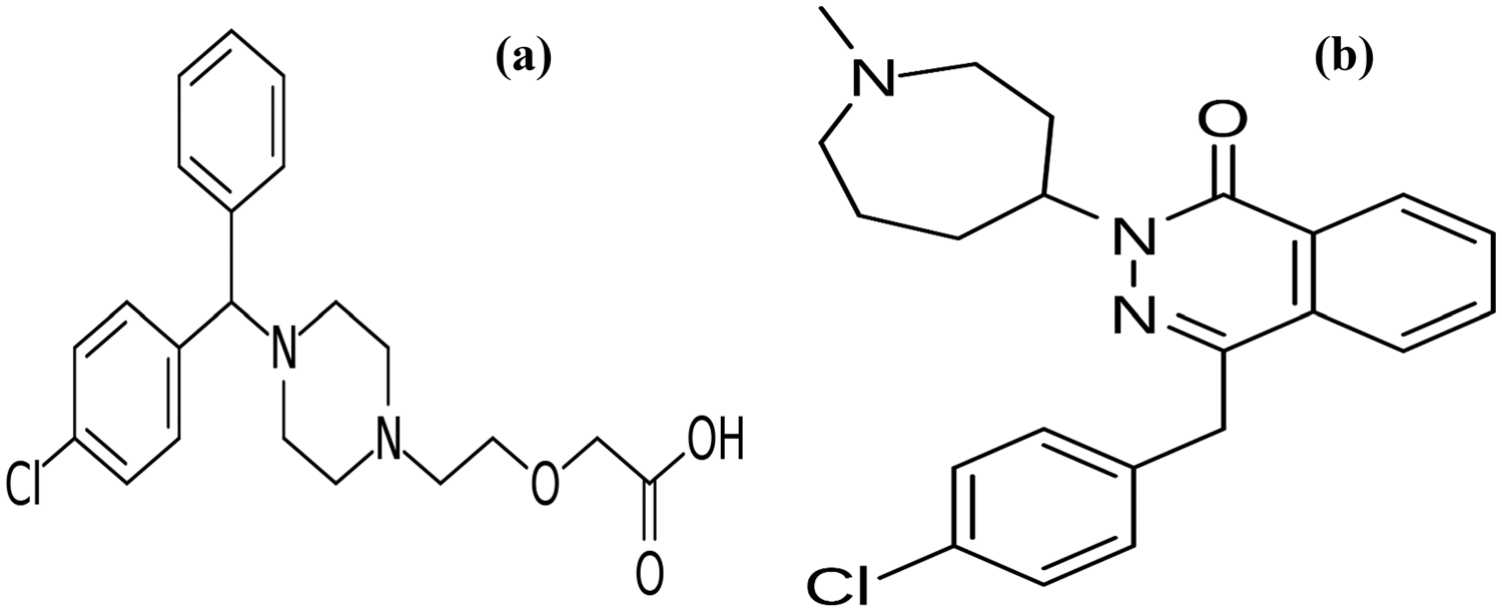
Structures of (**a**) cetirizine and (**b**) azelastine

CTZ is [2-[4-[(4-Chlorophenyl) phenylmethyl]-1–piperazinyl] ethoxy acetic acid) (Fig. 1a) [5]. It is histamine (H1)-receptor antagonist used in ocular allergy [6]. BP [5] recommended non aqueous potentiometric titration using sodium hydroxide while USP [7] stated HPLC for CTZ assay. There are several documented methods for its assay either in dosage form or human plasma as HPLC [8–12], capillary electrophoresis [13, 14], spectrofluorimetry [15–18] and spectrophotometry [19, 20].

AZE is 4-[(4-chlorophenyl)methyl]-2-(1-methylazepanyl)-1-phthalazinone (Fig. 1b) [5]. It is mast cell stabilizer that is used topically to relief allergic conjunctivitis [21]. Both BP [5] and USP [7] recommended non aqueous potentiometric titration with perchloric acid for AZE assay. Many articles have been published for its assay as HPLC [22–26], HPTLC [27, 28], capillary electrophoresis [29], electrochemical analysis [30, 31], NMR [32], spectrofluorimetry [33–35] and spectrophotometry [36–38].

Topical antihistamine and mast cell stabilizer are commonly co-administered for treatment of eye allergy [39]. CTZ as an antihistamine provides relief from symptoms before AZE starts working. AZE inhibits degranulation and the release of histamine. So, the effect of cetirizine in eye allergy can be enhanced by the addition of AZE [39].

To date, there is no documented method for the simultaneous determination of CTZ and AZE drug co-administration in aqueous humor. So, there is a need to establish a reliable new procedure for their assay in aqueous humor for therapeutic drug monitoring. The normal emission fluorescence spectra of the two studied drugs are severely overlapped when applying conventional spectrofluorimetric approach. Therefore, we applied synchronous fluorescence spectroscopy (SFS) to solve such problem and analyze each drug in the presence of the other in aqueous humor.

In constant wavelength SFS, both excitation and emission monochromators are simultaneously scanned at constant scan rates and a constant wavelength interval (∆λ) is kept between excitation and emission wavelengths. In conventional fluorescence, the intensity of fluorescence emission relies on emission wavelength while in SFS, it relies on ∆λ (both excitation and emission wavelengths). For constant wavelength SFS, the fluorescence intensity can be expressed as:

$$\text{F}=\text{klc}\ \text{Ex}({\lambda_{em}} - \Delta\lambda)\;{E_m}\;({\lambda_{em}})$$

Where c is the concentration of analyte, l is the path length of light and k is an experimental constant. For a given set of experimental conditions, fluorescence intensity is proportional to the concentration of the analyte. SFS is useful in the analysis of mixtures due to its apparent advantages including high selectivity, low scattering light interference, simple spectra and quick measurement in a single run [40].

Accordingly, a reproducible, sensitive, cheap, easy and eco-friendly SFS procedure was studied for the assay of CTZ and AZE simultaneously in their synthetic mixtures, aqueous humor as well as in their single eye drops. Assessment of the adopted method greenness was also performed using GAPI and analytical Eco-scale tools.

## Experimental

### Materials and Reagents

- CTZ and AZE pure samples (99.95% and 99.80% purity as labeled, respectively) were supplied by Apex Co. (Cairo) and European Egyptian Pharmaceuticals Industry (Alexandria), respectively.
- Ophthalmic formulations: Cetirizine^®^ eye drops 1%, a product of Pharo Pharma (batch no. 5669002). Azelast^®^ eye drops 0.05% manufactured by The Tenth of Ramadan for Pharmaceutical industries and diagnostic (RAMEDA) (for Hikma Pharmaceutical industries) (batch no. 202792).
- HPLC organic solvents were from Sigma Aldrich (Germany).
- Acetic acid, phosphoric acid, boric acid, sodium hydroxide, hydrochloric acid, sulfuric acid, nitric acid, tween 80, sodium dodecyl sulphate, β-cyclodextrin, carboxy methyl cellulose and cetrimide were all attained from El-Nasr Pharmaceutical Chemicals Co. (Cairo, Egypt).
- The water utilized throughout the experiment was double distilled water.

### Instruments

A Cary Eclipse spectrofluorometer with a xenon lamp and 5 mm slits was utilized. The synchronous mode was set at ∆λ = 60 nm and smoothing factor = 20. A Sonix IV model-SS101H 230 (USA) was utilized. A Consort pH meter was utilized for pH adjustment.

### Preparation of Standard Solutions

In 100 mL calibrated flasks, standard stock solutions (100 µg mL^−1^) of both CTZ and AZE were prepared separately by dissolving 10.0 mg of each in 100 mL methanol. Then, dilution was made to have standard working solutions of both drugs (10 µg mL^−1^) in methanol. All solutions were wrapped in aluminum foil [5] and kept in the refrigerator.

### Procedures

#### Construction of the Calibration Curves

In 10 mL calibrated flasks, appropriate volumes of CTZ and AZE standard working solutions were transferred separately to get final concentration ranges (0.1–2 µg mL^−1^) for both drugs. Then 1 mL 0.4 M H_2_SO_4_ was added followed by dilution to 10 mL with water and mixed well. A blank experiment was carried out in the same manner to obtain the relative synchronous fluorescence intensity (RSFI). The synchronous fluorescence spectra were measured at ∆λ = 60 nm. CTZ and AZE spectra were measured at 231 and 294 nm, respectively. Besides, RSFI were plotted versus the ultimate drugs concentrations (µg mL^−1^) and the regression equations were computed.

#### Analysis of CTZ/AZE in Laboratory Prepared Mixtures

Laboratory-prepared mixtures of CTZ and AZE in different ratios were prepared from the standard working solutions in 10 mL calibrated flasks. The procedure under **‘**Construction of the calibration curves’ was carried out.

#### Analysis of CTZ/AZE in their Ophthalmic Formulations

For Cetirizine^®^: one milliliter was taken from the formulation into 100 mL flask and the volume was completed with methanol. Then, five milliliters were transferred from the preceding solution into 50 mL flask (10 µg mL^−1^).

For Azelast^®^: one milliliter was taken into 50 mL flask and the volume was completed with methanol (10 µg mL^−1^). Samples in the linearity range were taken and the procedure described under **‘**Construction of calibration curves’ was applied to calculate the content of the eye drops from the regression equations.

#### Analysis of CTZ/AZE in Aqueous Humor

Artificial aqueous humor was prepared in the lab to simulate the natural one of that of the human [41]. In 10 mL calibrated flasks, one milliliter of the artificial aqueous humor was transferred. Subsequently, different volumes of both CTZ and AZE working standard solutions containing (1.0–20 µg) were added. Implement the steps mentioned in **‘**Construction of the calibration curves’.

## Results and Discussion

### Spectral Characteristics

**Fig. 2:**
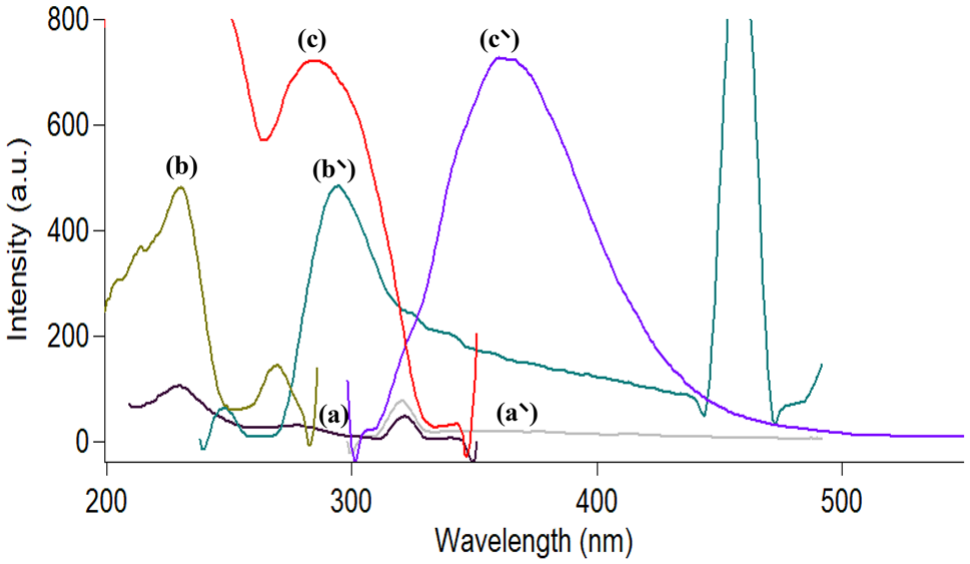
Excitation and emission spectra of: water using 0.4 M H_2_SO_4_ (blank) (**a**, **a’**), cetirizine (**b**, **b’**) and azelastine (**c**, **c’**) (concentration of each 2.0 µg ml^−1^)

**Fig. 3:**
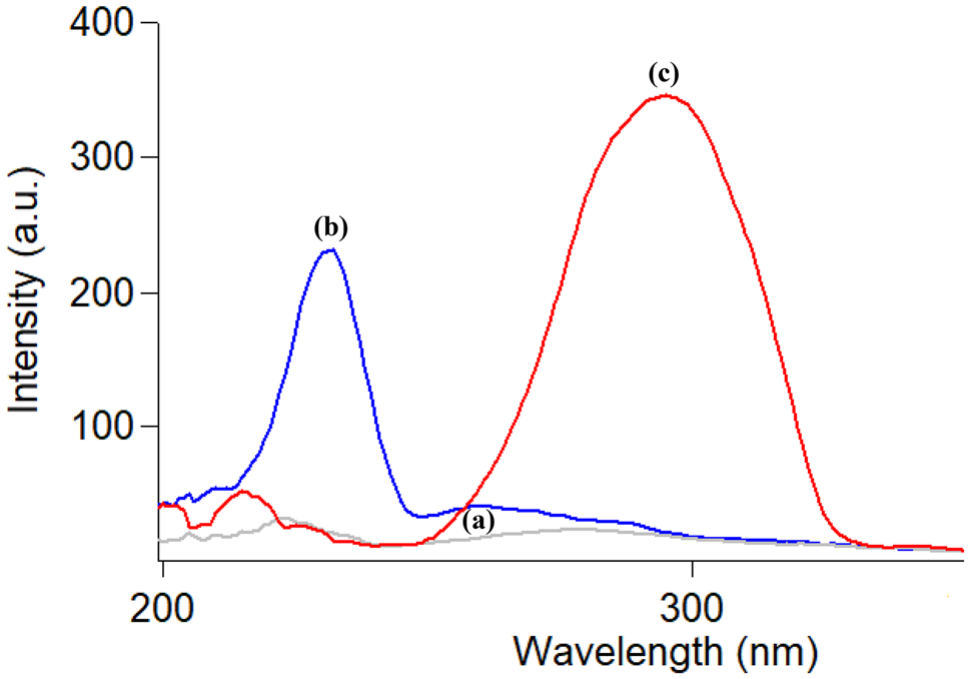
Synchronous fluorescence spectra of: (**a**) water using 0.4 M H_2_SO_4_ (blank), (**b**) cetirizine and (**c**) azelastine (concentration of each 1.0 µg ml^−1^)

Both CTZ and AZE were stated to exhibit innate fluorescence. CTZ was estimated by conventional spectrofluorimetry in acidic medium using water as the diluting solvent at 235/294 nm [18]. Also, AZE was estimated in water at 286/364 nm [34]. As shown in Fig. 2, it is noticed that the spectra of both drugs suffered from overlapping. To solve this problem, SFS mode was operated which resulted in a good separation of the two spectra permitting their assay in aqueous humor simultaneously. As shown in Fig. 3, CTZ could be estimated at 231 nm in presence of AZE, and AZE at 294 nm in presence of CTZ.

Table 1 showed a comparison of the adopted method to the published work for the assay of each drug.

**Table 1:** A comparison of the adopted method and some of the published methods

**Method**	**Matrix**		**Linearity**	**LOQ**	**Remarks**	**Ref**
**For cetirizine**						
HPLC-mass spectrometric detection	Human plasma		1–400 ng mL^−1^	1.0 ng mL^−1^	1. Gradient elution Water: formic acid 0.13% (solvent A) and methanol (solvent B) 2. Expensive detector	[8]
HPLC–UV	Pharmaceutical preparations		1–20 µg mL^−1^	1.0 µg mL^−1^	Acetonitrile: phosphate buffer pH 3.5 (40: 60 v/v)	[9]
HPLC–UV	Pharmaceutical preparations		5–50 µg mL^−1^	1.9 µg mL^−1^	Methanol: phosphate buffer pH 3.5 (80: 20 v/v)	[10]
HPLC–UV	Pharmaceutical preparations and human serum		2.5–50 µg mL^−1^	0.035 µg mL^−1^	Methanol: acetonitrile: water pH 3.1 (50: 20: 30 v/v/v)	[11]
HPLC–UV	Human serum		1–100 µg mL^−1^	2.0 µg mL^−1^	Methanol: water pH 2.8 (70: 30 v/v)	[12]
Spectrofluorimetry	Pharmaceutical preparation and human plasma		40–400 ng mL^−1^	8.3 ng mL^−1^	1. Reaction with potassium persulphate and 2-cynoacetamide in alkaline medium 2. 2-cyanoacetamde is an irritant reagent to the skin and eye	[15]
Spectrofluorimetry	Pharmaceutical preparations		3.5–129.3 µg mL^−1^	3.5 µg mL^−1^	Enhancement of rhodamine B-sodium tetraphenylborate reagent	[16]
Spectrofluorimetry	Pharmaceutical preparations		(1) 0.5–7 (2) 0.5–6 (3) 0.2–4 µg mL^−1^	(1) 0.48 (2) 0.17 (3) 0.45 µg mL^−1^	1. Charge transfer complexation in acetone with: (1) Dichloro-5,6-dicyano-1,4-benzoquinone (2) Tetracyanoethylene (3) p-chloranilic acid 2. Using acetone as organic solvent which is not green with high amounts	[17]
Spectrofluorimetry (comparison method)	Pharmaceutical preparations		0.1–2.0 µg mL^−1^	0.099	1. Using 4 mL 0.5 M perchloric acid and water as the diluting solvent 2. Perchloric acid is not a green reagent	[18]
**SFS (proposed method)**	Pharmaceutical preparations and with AZE in aqueous humor		0.1–2.0 µg mL^−1^	0.043 µg mL^−1^	Using 1 mL 0.4 M H_2_SO_4_ and water as the diluting solvent	
**For azelastine**						
HPLC–UV	Pharmaceutical preparations		0.2–20 µg mL^−1^	0.021 µg mL^−1^	Acetonitrile: phosphate buffer pH 3.5 (32:68 v/v)	[22]
HPLC–UV	Pharmaceutical preparations		6.25–50 µg mL^−1^	2.41 µg mL^−1^	Acetonitrile: phosphate buffer pH 4.5 (50:50 v/v)	[23]
Spectrofluorimetry	Pharmaceutical preparations		10–250 ng mL^−1^	4.61 ng mL^−1^	Using 0.2 M H_2_SO_4_ as the diluting solvent	[33]
Spectrofluorimetry (comparison method)	Pharmaceutical preparations		0.1–1.5 µg mL^−1^	0.073 µg mL^−1^	Using water as the diluting solvent	[34]
Spectrofluorimetry	Pharmaceutical preparations		2–40 µg mL^−1^	0.4845 µg mL^−1^	Using ethanol as the diluting solvent	[35]
**SFS (proposed method)**	Pharmaceutical preparations and with CTZ in aqueous humor		0.1–2.0 µg mL^−1^	0.029 µg mL^−1^	Using 1 mL 0.4 M H_2_SO_4_ and water as the diluting solvent	

### Optimization of SFS factors

#### Selection of Optimal Δ λ

The value of ∆λ is important in SFS as it affects peak resolution and sensitivity. Firstly, different ∆λ values (20–140 nm) were tested. From the study, ∆λ = 60 nm was the most suitable as it provided better peak shape and sensitivity for both drugs. Lower ∆λ values than 60 nm led to lower fluorescence intensity and higher ∆λ values than 60 nm led to poor separation of the two spectra.

#### Impact of pH

**Fig. 4:**
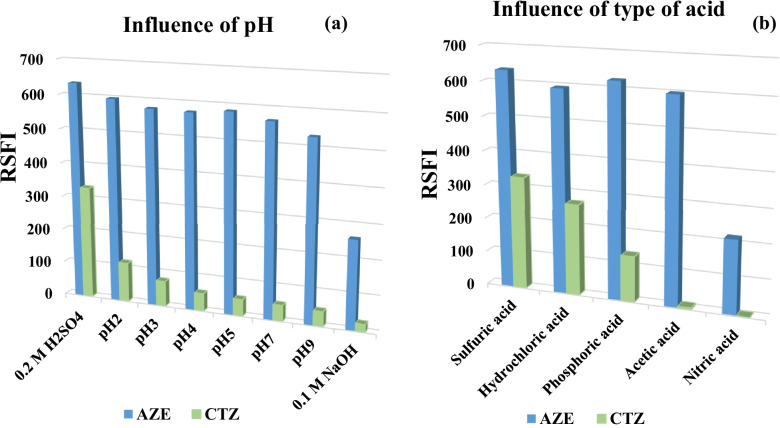
Influence of: (**a**) pH (**b**) type of acid on the synchronous fluorescence intensity of cetirizine and azelastine (concentration of each 2.0 µg ml^−1^)

Briton Robinson buffer with pH range (2–12), 0.2 M H_2_SO_4_ and 0.1 M NaOH were used to study the impact of pH on the RSFI. Both CTZ and AZE has nitrogen atom in their structures which could be protonated in acidic solutions. The protonation has shown to significantly increase fluorescence intensity [18, 42]. This may be the reason behind the increased RSFI of both drugs in more acidic solutions and decreased in basic solutions. In more basic solutions (0.1 M NaOH), RSFI decreased significantly which may be attributable to their degradation. However, RSFI enhanced in 0.2 M H_2_SO_4_ for both drugs especially CTZ (Fig. 4a). So, H_2_SO_4_ was chosen in the study to increase the sensitivity for both drugs.

#### Impact of Type and Concentration of Acid

Different acids were tried in the study. These acids were: nitric acid, acetic acid, phosphoric acid, hydrochloric acid and sulfuric acid. As shown in Fig. 4b, sulfuric acid was the most suitable one as it gave high RSFI for both compounds. Furthermore, the influence of H_2_SO_4_ concentration on RSFI of CTZ and AZE was studied. The results showed that increasing the molarity up to 0.3 M led to eventual increase in RSFI of CTZ, molarities 0.4 M and 0.5 M gave no increase in RSFI than 0.3 M. For AZE, H_2_SO_4_ concentration (0.05–0.5 M) had no significant effect on RSFI. So, H_2_SO_4_ concentration of 0.4 M was chosen in the SFS.

#### Impact of Surfactants

Different surfactants were utilized to check their impact on the RSFI (1% w/v of each). All the studied surfactants (carboxymethyl cellulose, cetrimide, sodium dodecyl sulphate, β-cyclodextrin, tween 80) led to decrease in RSFI for the two compounds when compared to using 0.4 M H_2_SO_4_. Hence, the study was continued without surfactant.

#### Impact of Diluting Solvents

Various solvent systems were attempted to determine the best conditions such as fluorescence intensity, stability and separation of the two spectra. These solvents were: water, methanol, ethanol, acetonitrile, isopropanol, butanol, dimethyl formamide and acetone. Butanol, isopropanol and dimethyl formamide gave high blank readings for both compounds. Acetone completely quenched the fluorescence intensity of the two drugs. Methanol, ethanol and acetonitrile caused shift in the maximum wavelength of CTZ and interference was noticed between the two compounds. Water was found to be the optimal one as it provided the best sensitivity and spectra separation. Also, it adds advantages to the procedure to be ecofriendly and cost effective without utilizing organic solvents.

#### Impact of Time

Time effect on the RSFI of CTZ and AZE was also studied. RFSI occurred immediately and remained stable for two hours.

### Validation of the Proposed Method

To confirm that the suggested SFS method is suitable for its intended use, the ICH Guidelines [43] were followed.

#### Linearity

A linear relationship was obtained between RSFI values and their corresponding drug concentrations (µg mL^−1^) over the range 0.1–2 µg mL^−1^ for both drugs. Linear analysis was made and the resulted regression equations were as following:

$$\mathrm{RSFI}\:=\:4.1054\:+\:205.5795\;\mathrm C\;(\mathrm r\:=\:0.9999)\;\mathrm{for}\;\mathrm{CTZ}\;\mathrm{at}\;231\;\mathrm{nm}$$

$$\mathrm{RSFI}\:=\:1.5033\:+\:327.5993\; \mathrm C\;(\mathrm r\:=\:0.9999)\;\mathrm{for\ AZE\ at}\;294\;\mathrm{nm}$$

Where C is the concentration of the drug in µg mL^−1^. The calculated analytical parameters are presented in Table 2.

**Table 2:** Validation data of the SFS method

**Parameter**	**Cetirizine**	**Azelastine**
∆ λ	60 nm	
Linearity range (µg mL^−1^)	0.1–2.0	0.1–2.0
Intercept (a)	4.1054	1.5033
Slope (b)	205.5795	327.5993
Correlation coefficient (r)	0.9999	0.9999
n	9	7
Standard deviation of residuals (S_y/x_)	1.6099	1.6697
Standard deviation of intercept (S_a_)	0.8769	0.9511
Standard deviation of slope (S_b_)	0.9902	1.0378
%Relative standard deviation (%RSD)	1.170	1.175
%Error	0.3910	0.4460
LOD (µg mL^−1^)	0.014	0.010
LOQ (µg mL^−1^)	0.043	0.029

#### Limit of Detection (LOD) and Limit of Quantification (LOQ)

LOD and LOQ were calculated in Table 2 pursuant to ICH Guidelines [43] by applying the following equations:

LOD = 3.3 σ/S and LOQ = 10 σ/S. Where σ: Standard deviation of the intercept. S: the slope of the regression line. LOD and LOQ values indicate the high sensitivity of the suggested method.

#### Accuracy and Precision

The accuracy was confirmed from the accepted percentage recoveries in Table 3. Also, the results were compared with those of the comparison methods [18, 34] and it was found that there is no remarkable difference between the methods. Intraday precision was performed by estimating three varied concentrations within the linearity range three times in the same day. But, interday precision was performed in three different days. The accepted % RSD values (Table 4) prove the precision.

**Table 3:** Analysis of cetirizine and azelastine in their raw materials by SFS method

	**Cetirizine**			**Azelastine**		
Parameters	Concentration taken (µg mL^−1^)	Concentration found (μg mL^−1^)	%found^*^	Concentration taken (μg mL^−1^)	Concentration found (µg mL^−1^)	%found^*^
	0.1	0.101	101.00	0.1	0.102	102.00
	0.2	0.203	101.50	0.2	0.203	101.50
	0.3	0.301	100.33	0.3	0.304	101.33
	0.5	0.510	102.00	0.5	0.499	99.80
	0.6	0.592	98.67	0.7	0.691	98.71
	0.7	0.695	99.29	1	0.997	99.70
	0.9	0.903	100.33	2	2.004	100.20
	1	0.987	98.70			
	2	2.006	100.30			
Mean ± S.D			100.24 ± 1.17			100.46 ± 1.18
	Comparison method (n = 4) [18]			Comparison method (n = 4) [34]		
Mean ± S.D	100.45 ± 1.49			100.04 ± 0.64		
*t*	0.25 (2.20) ^**^			0.77 (2.26) ^**^		
F	1.62 (4.07) ^**^			3.40 (4.76) ^**^		

^*^ Average of three replicate determinations

^**^ The theoretical t and F values (P = 0.05) are between parentheses[44]

**Table 4:** Precision data for the determination of CTZ and AZE by the studied method

		Intraday precision		Interday precision	
Concentration (µg mL^−1^)		Mean ± S.D	%RSD	Mean ± S.D	%RSD
Cetirizine	0.2	99.68 ± 1.86	1.87	99.33 ± 1.88	1.89
	0.5	99.71 ± 1.01	1.01	100.26 ± 1.22	1.22
	1	98.34 ± 0.31	0.32	98.22 ± 0.41	0.42
Azelastine	0.2	100.63 ± 0.92	0.92	100.83 ± 1.42	1.41
	0.5	99.20 ± 0.61	0.61	99.00 ± 0.71	0.72
	1	99.37 ± 0.54	0.54	100.05 ± 0.41	0.41

#### Selectivity

**Fig. 5:**
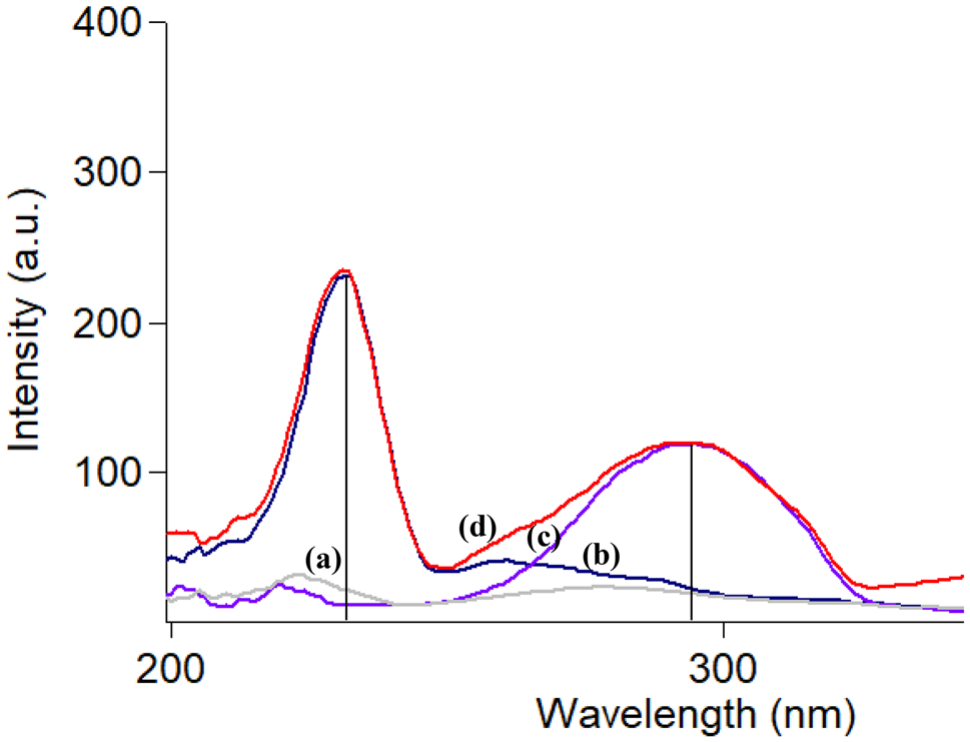
Synchronous fluorescence spectra of: (**a**) water using 0.4 M H_2_SO_4_, (**b**) 1 µg mL^−1^ cetirizine, (**c**) 0.3 µg mL^−1^ azelastine and (**d**) a mixture of 1 µg mL^−1^ cetirizine and 0.3 µg mL^−1^ azelastine

**Fig. 6:**
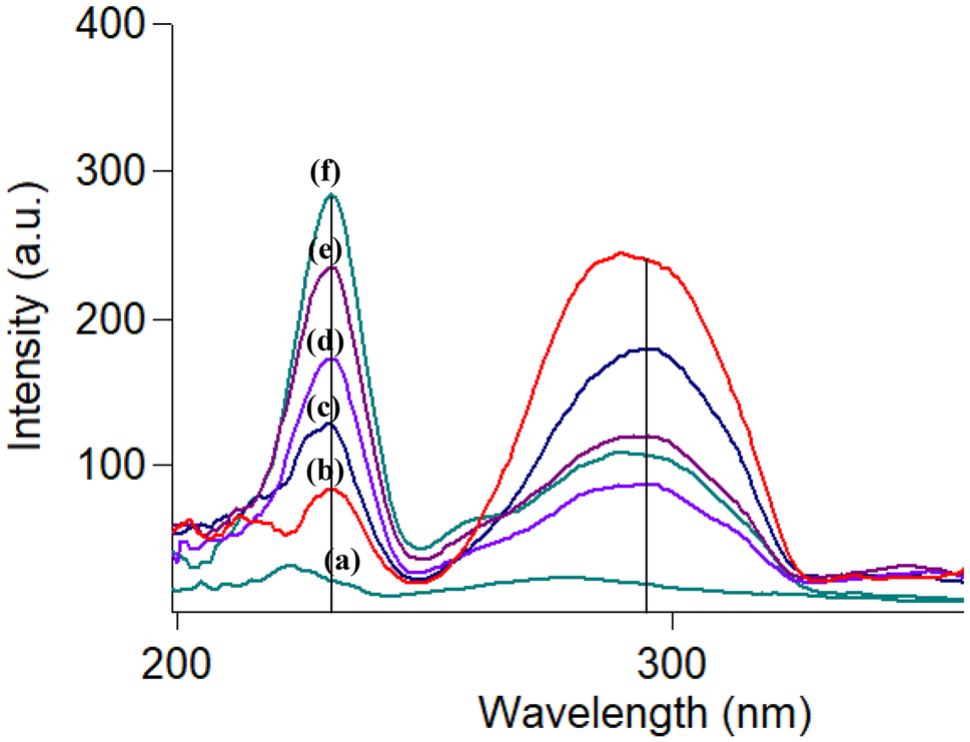
Synchronous fluorescence spectra of: (**a**) blank. (**b**-**f**) Concentrations of drugs in aqueous humor (0.3 + 0.7 µg mL^−1^), (0.5 + 0.5 µg mL^−1^), (0.7 + 0.2 µg mL^−1^), (1 + 0.3 µg mL^−1^) and (1.2 + 0.25 µg mL^−1^) of cetirizine and azelastine, respectively

The SFS procedure was used for the synchronized estimation of CTZ and AZE in synthetic combinations composed of different ratios of the two drugs as shown in Fig. 5. CTZ was measured at 231 nm where AZE shows no interference. Similarly, AZE was measured at 294 nm without any interference from the other drug. Upon measuring the peak amplitude of each drug, the corresponding drug concentrations were calculated from the regression equation. Satisfactory results were obtained and mentioned in Table 5. Furthermore, the method selectivity was achieved by the estimation of the studied drugs in aqueous humor. The prepared artificial aqueous humor composed of sodium chloride, potassium chloride, dibasic sodium phosphate, sodium bicarbonate, calcium chloride, potassium chloride dihydrate, magnesium chloride hexahydrate, dibasic sodium phosphate, sodium bicarbonate, dextrose, glutathione disulfide, hydrochloric acid and/or sodium hydroxide and water for injection [41]. None of these components interfered with the analysis of the two compounds as proved by the high percentage recoveries and the small values of SD for CTZ and AZE assay in aqueous humor (Table 6 and Fig. 6).

**Table 5:** Analysis of CTZ and AZE in their synthetic mixtures by the studied method

	Concentration taken (µg mL^−1^)	Concentration taken (µg mL^−1^)	Concentration found (µg mL^−1^)	Concentration found (µg mL^−1^)	%found^*^	%found^*^
Mix. no	CTZ	AZE	CTZ	AZE	CTZ	AZE
1	0.5	0.5	0.496	0.496	99.13	99.20
2	0.7	0.2	0.688	0.204	98.26	101.95
3	1.2	0.25	1.220	0.255	101.70	101.95
4	1	0.3	1.011	0.304	101.13	101.24
5	2	0.1	1.960	0.100	97.99	100.72
Mean ± S.D					99.64 ± 1.68	101.01 ± 1.14
%RSD					1.69	1.13
%Error					0.76	0.50

^*^ Average of three replicate determinations

**Table 6:** Application of the studied SFS method for the determination of CTZ and AZE in spiked aqueous humor

Parameter	Concentration taken (µg mL^−1^)	Concentration taken (µg mL^−1^)	Concentration found (µg mL^−1^)	Concentration found (µg mL^−1^)	%found	%found
	**CTZ**	**AZE**	**CTZ**	**AZE**	**CTZ**	**AZE**
	0.3	0.7	0.294	0.690	97.93	98.55
	0.5	0.5	0.496	0.495	99.13	99.02
	0.7	0.2	0.710	0.198	101.38	99.20
	1	0.3	0.980	0.299	97.96	99.71
	1.2	0.25	1.225	0.251	102.11	100.36
Mean ± S.D					99.83 ± 1.95	99.37 ± 0.69
%RSD					1.95	0.70
%Error					0.87	0.40

#### Robustness

Assessment of the suggested method robustness was performed by applying premeditated slight variation in the concentration of H_2_SO_4_ (0.4 M ± 0.1)_._ These variations had negligible influence on RSFI which reflects the robustness.

#### Applications

##### Assay of CTZ/AZE Synthetic Mixtures

The suggested procedure was implemented for the estimation of the investigated compounds in their synthetic mixtures (Fig. 5). The accepted %recoveries reflect the accuracy (Table 5).

##### Assay of CTZ and AZE in their Ophthalmic Formulations

This synchronous spectrofluorimetry was utilized to estimate CTZ and AZE directly in their ophthalmic formulations for extending its utilization in quality control laboratories. The results attained were contrasted with those of the comparison procedures as presented in Table 7. Statistical assessment of the results utilizing F and t tests demonstrated that there is no remarkable difference between the two procedures [44].

**Table 7:** Analysis of cetirizine and azelastine in their ophthalmic formulations by the proposed and comparison methods

	Proposed method			Comparison methods [18, 34]
Ophthalmic formulations	Concentration taken (µg mL^−1^)	Concentration found (µg mL^−1^)	%found^*^	%found^*^
Cetirizine^®^ 1%	0.2	0.203	101.89	102.00
	0.5	0.509	101.95	99.00
	1.0	1.001	100.15	99.00
				100.80
Mean ± S.D			101.33 ± 1.02	100.20 ± 1.47
%RSD			1.01	1.47
*t*	1.20 (2.57) ^**^			
F	2.08 (19.16) ^**^			
Azelast^®^ 0.05%	0.2	0.197	98.44	98.50
	0.7	0.691	98.77	100.80
	1.0	0.980	98.02	99.80
Mean ± S.D			98.41 ± 0.38	99.70 ± 0.15
%RSD			0.38	1.16
*t*	1.84 (2.78) ^**^			
F	9.16 (19.00) ^**^			

^*^ Average of three replicate determinations

^**^ The theoretical t and F values (P = 0.05) are between parentheses [44]

##### Assay of CTZ and AZE in Aqueous Humor

The suggested procedure enables the determination of CTZ and AZE simultaneously in aqueous humor (Fig. 6). Data in Table 6 revealed that the mean absolute recoveries and % RSD of CTZ and AZE in aqueous humor were 99.83 ± 1.95 and 99.37 ± 0.69, respectively.

##### Assessment of the Greenness of the Proposed Method

**Fig. 7:**
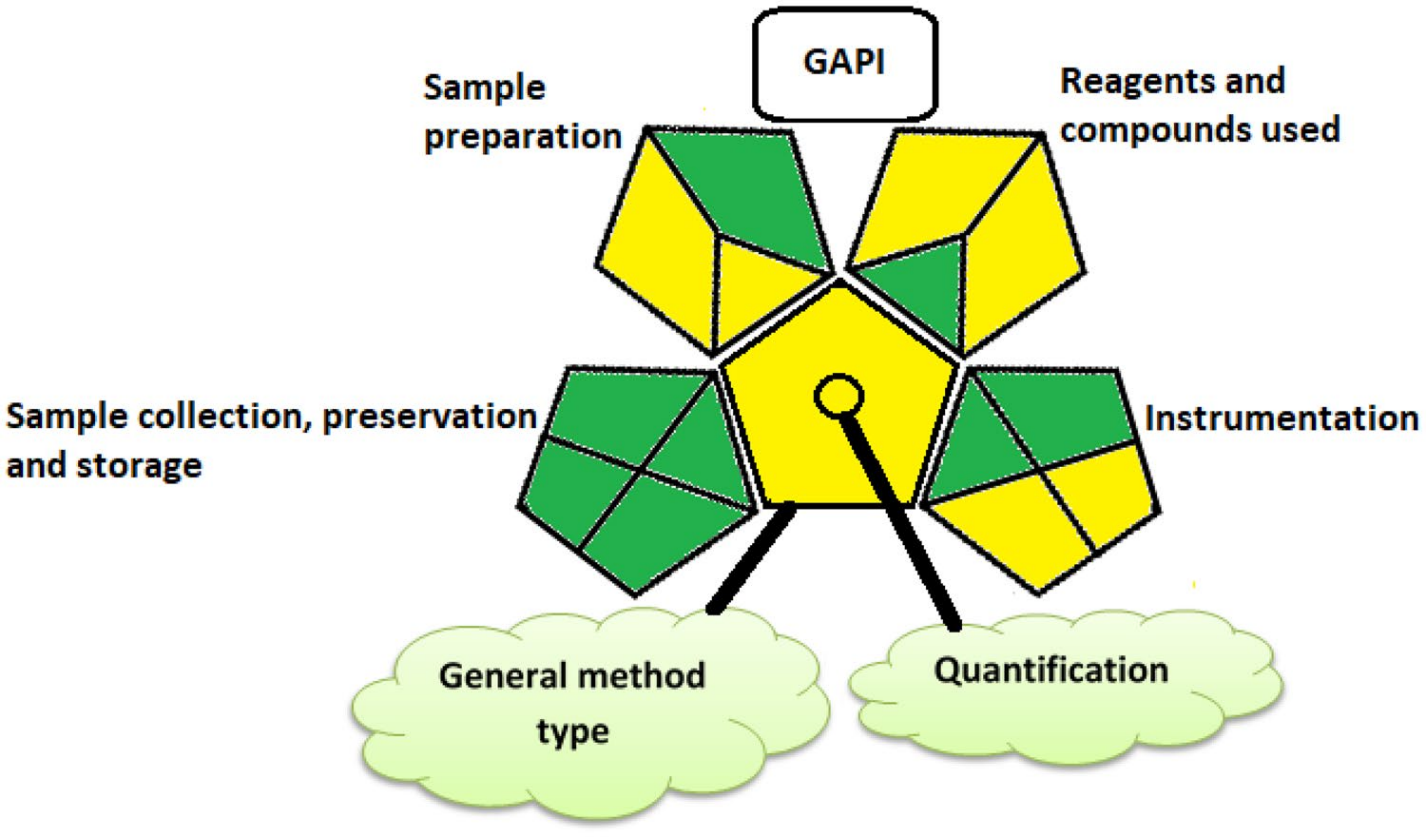
The green assessment profile for the proposed SFS using the GAPI tool [45]

Several analytical tools are currently present to assess the methodologies concerning their ecological impact. GAPI and analytical Eco-scale were conducted in this study. The viewable presentation of GAPI (five pentagram) makes it easy to select the greenest approach for a definite study [45]. It is a semi-quantitative tool that gives exhaustive information on the evaluated practices through providing a more detailed evaluation for each step of the analytical methodology from sample collection to final determination. GAPI assessment tool of the studied procedure is shown in Fig. 7.

Analytical Eco-scale [46] is another greenness assessment tool. It determines penalty points for various factors involved in the study. Then, the total score is subtracted from the ideal value 100. The suggested procedure is found to be an excellent green one (Table 8).

**Table 8:** Analytical Eco-scale penalty points [46] of the proposed SFS approach

**Reagents**	**Penalty points**
Water	0
1 mL of 0.4 M H_2_SO_4_	2
	∑2
**Instruments**	**Penalty points**
Spectrofluorimeter	0
Occupational hazard	0
Waste	3
	∑3
Total penalty points	5
**Score**	**95**

## Conclusion

In severe ocular allergies, a combination of cetirizine and azelastine eye drops could be recommended. A green, easy and quick synchronous fluorescence approach was studied for the first time to estimate both drugs simultaneously in aqueous humor for clinical assessment. The method was simple and did not require the use of expensive equipment or solvents. The method was subjected to ICH guidelines and characterized by wide linearity range, accuracy, precision, selectivity and robustness. Furthermore, it could be useful for analyzing the cited drugs in their ophthalmic formulations in quality control laboratories.

## Data Availability

All the data and the materials are available all-over the study.

## Code Availability

No software or any computational study was not used for this study.
